# Alpha-Mangostin Attenuation of Hyperglycemia-Induced Ocular Hypoperfusion and Blood Retinal Barrier Leakage in the Early Stage of Type 2 Diabetes Rats

**DOI:** 10.1155/2015/785826

**Published:** 2015-04-09

**Authors:** Amporn Jariyapongskul, Chonticha Areebambud, Sunit Suksamrarn, Chantana Mekseepralard

**Affiliations:** ^1^Department of Physiology, Faculty of Medicine, Srinakharinwirot University, Bangkok 10110, Thailand; ^2^Biomedical Science Program, Faculty of Medicine, Srinakharinwirot University, Bangkok 10110, Thailand; ^3^Department of Chemistry, Faculty of Science, Srinakharinwirot University, Bangkok 10110, Thailand; ^4^Department of Microbiology, Faculty of Medicine, Srinakharinwirot University, Bangkok 10110, Thailand

## Abstract

The present study examined effects of alpha-mangostin (*α*-MG) supplementation on the retinal microvasculature, including ocular blood flow (OBF) and blood-retinal barrier (BRB) permeability in a type 2 diabetic animal model. Male Sprague-Dawley rats were divided into four groups: normal control and diabetes with or without *α*-MG supplementation. Alpha-mangostin (200 mg/Kg/day) was administered by gavage feeding for 8 weeks. The effects of *α*-MG on biochemical and physiological parameters including mean arterial pressure (MAP), OBF, and BRB leakage were investigated. Additionally, levels of retinal malondialdehyde (MDA), advance glycation end products (AGEs), receptor of advance glycation end products (RAGE), tumour necrosis factor alpha (TNF-*α*), and vascular endothelial growth factor (VEGF) were evaluated. The elevated blood glucose, HbA1c, cholesterol, triglyceride, serum insulin, and HOMA-IR were observed in DM2 rats. Moreover, DM2 rats had significantly decreased OBF but statistically increased MAP and leakage of the BRB. The *α*-MG-treated DM2 rats showed significantly lower levels of retinal MDA, AGEs, RAGE, TNF-*α*, and VEGF than the untreated group. Interestingly, *α*-MG supplementation significantly increased OBF while it decreased MAP and leakage of BRB. In conclusion, *α*-MG supplementation could restore OBF and improve the BRB integrity, indicating its properties closely associated with antihyperglycemic, antioxidant, anti-inflammatory, and antiglycation activities.

## 1. Introduction

When the pancreas does not produce sufficient insulin or the body is unable to utilize the insulin, diabetes mellitus is developed. All forms of diabetes are characterized by chronic hyperglycemia. Long-lasting hyperglycemia leads to serious damage to organs and tissues of the body, including the vasculatures. The vascular complications of diabetes are associated with microvascular and macrovascular diseases which are retinopathy, nephropathy, cardiovascular disease, and cerebrovascular disease. Diabetic retinopathy (DR) is one of the most common microvascular complications of type 1 and type 2 diabetes leading to visual impairment and blindness [1, 2]. The major characteristics of DR developed during the progress of the disease include breakdown of blood retinal barrier (BRB), thickening of vascular basement membrane, pericytes loss, nonperfused vessels, microaneurysms, hemorrhage, and cotton-wool spots [3, 4]. It is now widely accepted that prolonged exposure to hyperglycemia induces an increase in oxidative stress, which plays an important role in the early and late stage of DR [5]. Several studies have reported that the origins of oxidative stress in diabetes are free radicals generated by several biochemical pathways. These pathways refer to polyol pathway [6], nonenzymatic glycation and advanced glycation end products (AGEs) [7], diacylglycerol-protein kinase C (DAG-PKC) [8], and hexosamine pathways [9].

Recently, accumulation of AGEs under hyperglycemic conditions has been of much interest to play an important role in the development of DR. AGEs have also been demonstrated to bring on oxidative stress production in various types of cells through the interaction with a receptor for AGEs (RAGE). Subsequently, the AGEs-RAGE induced the genesis of inflammatory reactions, leading to diabetic vascular complication [10, 11]. Moreover, the increase in AGEs could activate many downstream signaling molecules, including reactive oxygen species (ROS) and nuclear factor kappa B (NF-*κ*B) that induced inflammatory mediator productions such as adhesion molecules, interleukins, and tumor necrosis factor alpha (TNF-*α*) [12, 13]. There were many evidences to show the effect of AGEs on endothelial cells (ECs). Apart from increasing in leukocyte adhesion to cultured retinal microvascular endothelial cells [14], AGEs were able to decrease endothelial nitric oxide synthase (NOS) mRNA levels in ECs [15]. The AGEs also reduced nitric oxide (NO) bioavailability by inactivating NO to form peroxynitrite via ROS generation [16]. These evidences indicated that AGEs would be one of the important proinflammatory factors and a source of ROS induction for the progression of DR.

The target management for vascular complications of diabetes has been pointed out to inhibit inflammation and oxidative stress pathway. Presently, the herbal medicine has increasingly gained attention as a protector of diabetic vascular complications. Alpha-mangostin (*α*-MG), a yellow coloring agent, is the first xanthone isolated from bark and dried sap of mangosteen (*Garcinia mangostana*) [17]. This substance has a wide spectrum of biological actions including anti-inflammation and antioxidant properties. Williams et al. found that *α*-MG decreased human low-density lipoprotein (LDL) oxidation induced by peroxy-radical [18]. A decrease in *α*-tocopherol consumption induced by LDL-oxidation was inhibited by *α*-MG [19]. More recently, Buelna-Chontal et al. found that several xanthones isolated from the pericarp of* Garcinia mangostana* containing *α*-MG were able to scavenge peroxynitrite anion (ONOO^−^) [20]. In addition to antioxidant properties,* Garcinia mangostana* had an anti-inflammatory effect. Both histamine release and prostaglandin E_2_ (PGE_2_) synthesis in rat glioma cells were potently inhibited by 40% ethanol extract of mangosteen [21]. Chen et al. demonstrated that the *α*-MG and *γ*-mangostin (*γ*-MG) significantly inhibited lipopolysaccharide- (LPS-) stimulated peroxynitrite radical production, suggesting anti-inflammatory and antioxidant effects [22].

No* in vivo* study has been carried out to investigate the efficacy of *α*-MG to prevent or delay diabetic-induced retinal microangiopathy. In this study, we investigated effects of *α*-MG on diabetic retinopathy with reference to restoration of OBF detected by a laser Doppler flowmetry [23] and an improvement of BRB integrity evaluated by the Evans blue dye techniques [24] in type 2 diabetic rats. The study also included its antihyperglycemic, antioxidant, antiglycation, and anti-inflammatory activities, based on physiologic and metabolic parameters and determination of MDA, AGEs, RAGE, VEGF, and TNF-*α*, measured. The type 2 diabetic rat model used was induced by high fat (HF) diet feeding combined with a low dose streptozotocin (STZ) injection. This model was recently used as an alternative animal model for type 2 diabetes for impaired insulin secretion, glucose intolerance, insulin resistance, and obesity [25].

## 2. Materials and Methods

### 2.1. Alpha-Mangostin (*α*-MG) Preparation

The fruit mangosteen was collected from Kombang District, Chanthaburi Province, Thailand, in 2007. A voucher specimen (Porntip Wongnapa number 002) is deposited at the Faculty of Science, Ramkhamhaeng University, Bangkok, Thailand. Mangosteen extract was prepared by extracting dried and pulverized fruit pericarp (0.5 kg) in 95% ethyl alcohol (3 L) at room temperature for 48 hours. The solvent was then removed to give a brownish residue. Water was then added and the resulting yellow solid was separated and dried in vacuum to give a crude extract (35 g). The *α*-MG was isolated and purified by repetitive column chromatography as previously described [26]. Its purity exceeded 95% as determined by HPLC [27]. The alpha-mangostin at the concentration of 200 mg/kg BW of rat was freshly dissolved in 1.0 mL of corn oil (Mazola, Malaysia) before use.

### 2.2. Induction of Experimental Type 2 Diabetic Rat Model (DM2)

Type 2 diabetic rat model was induced as described previously [25]. Rats were daily fed with high fat diet consisting of 33.90% fat, 30.35% carbohydrate, and 20.50% protein with a total calorific value of 5,085 kcal/kg diet. After 3 weeks of dietary manipulation, the rats were intraperitoneally (IP) injected with streptozotocin (STZ; Sigma, St. Louis, MO, USA; 35 mg/kg, in 10 Mm sodium citrate buffer, pH 4.5). The normal control groups with or without *α*-MG treatment were given regular diet which contained 4.5% fat and 24% protein with a total calorific value of 3,040 kcal/kg diet for three weeks followed by an IP injection of 0.5 mL of 10 mM sodium citrate buffer, pH 4.5. Fasting blood glucose (FBG) level was determined on 7 days after STZ or citrate buffer injection. The rats with a FBG ≥ 250 mg/dL were diagnosed as diabetes mellitus.

### 2.3. Animals and Designs

Fifty-six male Sprague-Dawley rats at the age of 8 weeks (National Laboratory Animal Centre of Salaya Campus, Mahidol University, Thailand), weighing 150–180 g, were used in this study approved by the Ethical Committee, Faculty of Medicine, Srinakharinwirot University, Thailand (identification number EC 5/2556 SWU-MED). After a week of adaptation and housing under constant temperature of 20 ± 2°C at the Animal Centre, Faculty of Medicine, Srinakharinwirot University, Bangkok, Thailand, the rats were randomly divided into two main groups. Group A was designed for evaluating physiological and metabolic parameters, for example, serum insulin, ocular blood flow (OBF), and malondialdehyde (MDA, an end product of lipid peroxidation), advanced glycation end products (AGEs), receptor of glycation end products (RAGE), tumour necrosis factor alpha (TNF-*α*), and vascular endothelial growth factor (VEGF) in retinal tissue. Group B was monitored to study blood retinal barrier (BRB). Each main group was further divided into 4 subgroups of 7 rats. Normal control group (CON) was fed with regular diet for 8 weeks. Alpha-mangostin treated normal control group (CON-MG) was fed with regular diet combined with *α*-MG supplementation (200 mg/kg BW/day) for 8 weeks. Type 2 diabetic group (DM2) induced by high fat diet feeding combined with an IP injection of STZ was continuously fed with high fat diet for 8 weeks. Alpha-mangostin treated DM2 group (DM2-MG) was prepared in the same way as the DM2 group, but they also received gavage feeding of *α*-MG (200 mg/kg BW/day) during 8 weeks after STZ injection. To monitor all parameters, rats were anesthetized with an injection of sodium pentobarbital (60 mg/kg BW) and kept warm at 37°C on electric warming board. Their femoral veins and arteries were cannulated with polyethylene tubes for sodium pentobarbital administration to maintain anesthesia and for monitoring arterial blood pressure, respectively.

### 2.4. Determination of Physiologic and Metabolic Parameters

The physiologic and metabolic parameters were determined in all groups. Rat body weight was measured every two weeks after STZ or sodium citrate buffer injection until the end of experiment. Metabolic parameters including blood glucose, plasma hemoglobin A1c (HbA1c), serum insulin, and blood cholesterol and triglyceride were evaluated every 4 weeks after STZ or sodium citrate buffer injection. Blood glucose content was determined using blood glucose meter (Accutrend GCT, Roche, Germany) whereas blood cholesterol and triglycerides levels were analysed using enzymatic method (Professional Laboratory Management Corp. Co., Ltd, Thailand). Plasma HbA1c and serum insulin levels were determined using turbidimetric immunoinhibition method (Professional Laboratory Management Corp. Co., Ltd, Thailand) and Sandwich ELISA kit (Millipore, USA), respectively, by following the manufacturers' protocols. The blood glucose and serum insulin levels were used to calculate the insulin resistance using the homeostatic model assessment of insulin resistance (HOMA-IR) according to the following formula: HOMA-IR = (Glucose × Insulin)/22.5 [28]. Insulin is expressed as international dosage units per liter (U/L) and glucose is given in mmol/L.

### 2.5. * In Vivo* Measurement of the Ocular Blood Flow (OBF)

The left OBF was measured by using a laser Doppler flowmetry (moorLAB monitor, Moor Instruments, Ltd., England) with an optic needle probe of 0.1 mm diameter. The needle probe was fixed perpendicularly above the eye at about 1 mm from the surface of the cornea. Five different measurement points around the eye were performed on each rat, and the mean of five points of ocular blood flow was recorded. The data of OBF was expressed in arbitrary “perfusion units” (PU).

### 2.6. Preparation of Rat Retinal Homogenate

The eyeball of rat was removed and the retina was dissected under an operating microscope. The retinal tissue was homogenized for 90 seconds in 500 *µ*L of RIPA buffer (Sigma-Aldrich, St. Louis, MO, USA) plus 1% of protease inhibitor cocktail (Sigma-Aldrich, St. Louis, MO, USA). After clarifying by centrifugation for 15 minutes at 12,000 ×g, 4°C, the homogenate was collected and stored at −80°C until use. The supernatant aliquots were used for the quantification of the MDA, AGEs, RAGE, TNF-*α*, and VEGF.

### 2.7. Determination of Malondialdehyde, Advanced Glycation End Products, Receptor of Advanced Glycation End Products, Tumour Necrosis Factor Alpha, and Vascular Endothelial Growth Factor

The level of malondialdehyde was determined by thiobarbituric acid reactive substance (TBARs) assay in a form of MDA-TBA complex as described in the manufacturer's instruction (Cayman Chemical, USA). Quantitative sandwich enzyme immunoassays were used to measure rat AGEs (Cusabio, USA), RAGE (AbCam, UK), TNF-*α* (R&D System, USA), and VEGF (R&D System, USA) by following the manufacturer's protocols.

### 2.8. Determination of Blood Retinal Barrier (BRB) Permeability

In group B rat, blood brain barrier permeability was carried out using Evans blue (EB) technique. Evans blue dye solution used was prepared by dissolving the dye in normal saline (0.9% NaCl) to the concentration of 30 mg/mL. At the end of the experiment, the rat was fasted for 12 hours and anesthetized by IP injection of sodium pentobarbital (60 mg/kg BW). The femoral vein and femoral artery were cannulated each with a polyethylene tube and filled with heparinized saline (400 unit heparin/mL of 0.9% NaCl). EB solution was injected through the femoral vein over 10 seconds at a concentration of 45 mg/kg body weight. When the rat turned visibly blue, this confirmed its uptake and distribution of the dye throughout the body. After the dye had circulated for 120 minutes, the rat chest cavity was opened and warmed (37°C) normal saline was then perfused via the left ventricle at a physiological pressure of 120 mmHg for two minutes. The eyeball was enucleated and the retina was dissected under an operating microscope and weighed. To quantify EB leakage, the dye was extracted by incubating each retina in 300 *µ*L formamide (Sigma-Aldrich, St. Louis, MO) for 18 hours at 70°C. The extract was then centrifuged at 3,000 ×g for 1 hour, and 100 *µ*L of the supernatant was used to measure the optical density by a spectrophotometer at 620 nm. The concentration of EB dye in the extract was calculated from a standard curve of EB in formamide. BRB breakdown was calculated using the following formula: BRB permeability = EB (*µ*g)/weight of retina (mg).

### 2.9. Statistical Analysis

Results were presented as mean ± standard error of mean (SEM). Significant differences between groups were determined using one-way analysis of variance (one-way ANOVA), and differences between pairs of mean values were evaluated by the least significant difference (LSD) test. Statistical analysis was performed using SPSS IBM Singapore Pte Ltd. (registration number 1975-01566-C). A value of *P* < 0.05 was considered statistically significant.

## 3. Results

### 3.1. Biochemical and Physiological Parameters

**Figure 1:**
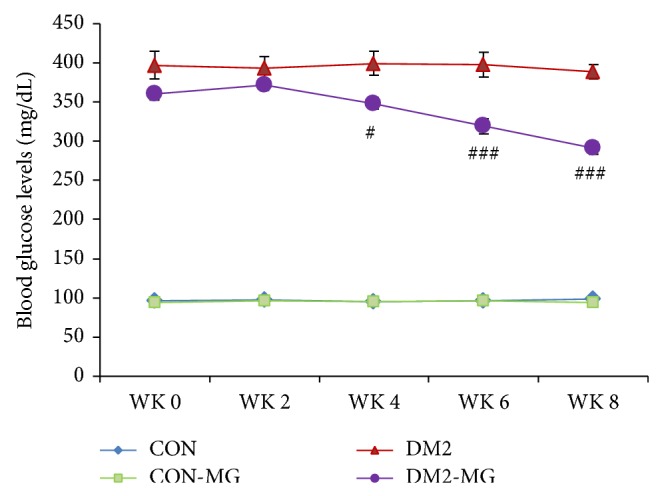
Fasting blood glucose (FBG) levels measured from the week 0 to week 8. CON: normal control rats; DM2: type 2 diabetic rats; CON-MG: *α*-MG supplemented normal control rats; DM2-MG: *α*-MG supplemented type 2 diabetic rats. Values are presented as mean ± SEM. ^#,###^ indicate significant differences compared to the DM2 rats at *P* < 0.05 and *P* < 0.001, respectively.

The antihyperglycemic potential of *α*-MG was shown in Figure 1. The fasting blood glucose (FBG) levels were monitored at the beginning of the study (WK 0) and every 14 days after STZ or vehicle injection or *α*-MG supplementation. Figure 1 showed changes in the mean levels of FBG in four groups (normal control rat: CON; type 2 diabetic rats: DM-2; *α*-MG supplemented rats: CON-MG rats; and *α*-MG supplemented type 2 diabetic rats: DM2-MG). We noted that the FBG levels in DM2 rats remained significantly high throughout the experimental period. Four weeks after supplementation of *α*-MG, although the FBG level of DM2-MG was mildly different from that of DM2 rats, it showed a significant reduction (*P* < 0.05). This reduction remained constant at the end of the experiment, exhibiting a strongly significant difference compared with the FBG level of DM2 group (*P* < 0.001).

The effects of *α*-mangostin on changes of physiological and biochemical parameters including mean arterial blood pressure (MAP), plasma glycated hemoglobin (HbA1C), serum insulin (S. insulin), homeostatic model assessment of insulin resistance (HOMA-IR), blood cholesterol (CHOL), and triglyceride (TG) in four groups of rats were summarized in Table 1. As expected, all the parameters tested in DM2 rats exhibited markedly higher levels than those of the CON rats. Interestingly, supplementation of *α*-MG for eight weeks had a strong potential to significantly reduce MAP, plasma HbA1C, serum insulin, blood cholesterol, and triglyceride and HOMA-IR, compared with those monitored from untreated DM2 rats. However, *α*-MG supplementation had no effect on changes of physiological and biochemical tests of nondiabetic rats.

### 3.2. The Effects of *α*-Mangostin on Ocular Blood Flow (OBF) Perfusion and Blood Retinal Barrier (BRB) Leakage

**Figure 2:**
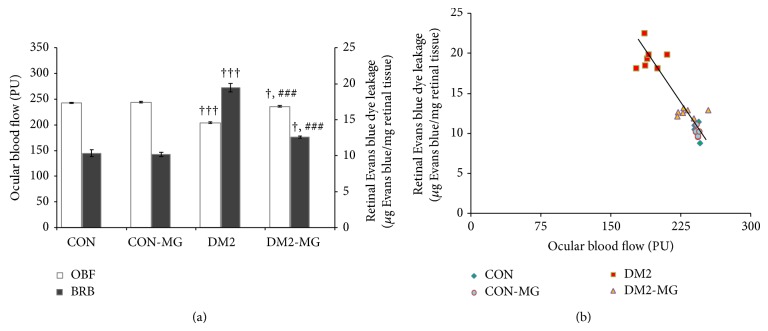
(a) Ocular blood flow (OBF) and retinal Evans blue dye leakage (blood retinal barrier leakage; BRB) and (b) correlation between OBF and BRB which were monitored in normal control rats (CON), type 2 diabetic rats (DM2), *α*-MG supplemented normal control rats (CON-MG), and *α*-MG supplemented type 2 diabetic rats (DM2-MG). Values are presented as mean ± SEM. ^+,+++^ indicate significant differences compared to the CON rats at *P* < 0.05 and *P* < 0.001, respectively. ^###^ indicates significant difference compared to the DM2 rats at *P* < 0.001. The linear regression equation was expressed as *y* = −0.157*x* + 48.8 (*R* ^2^ = 0.831), where *x* is ocular blood flow, *y* is blood retinal barrier leakage, and *R* is correlation coefficient.

Figures 2(a) and 2(b) showed the OBF perfusion measured using laser Doppler flowmetry and blood retinal barrier permeability evaluated using Evan blue (EB) dye technique. The mean OBF perfusion was reduced significantly in the DM2 rats compared with that of the control rats (*P* < 0.001), (Figure 2(a)). Surprisingly, the OBF were obviously recovered to around the level of the control rats after eight-week supplementation with *α*-MG (*P* < 0.001). The DM2 rats had significantly higher BRB leakage than the control rats (*P* < 0.001) (Figure 2(a)). However, the leakage was suppressed significantly by *α*-MG treatment (*P* < 0.001). Again, supplementation of *α*-MG had no effect on changes of mean OBF perfusion and leakage of BRB in control rats. Values of blood retinal barrier leakage and ocular blood flow perfusion were also plotted in Figure 2(b). The graph revealed a good correlation between ocular blood flow and blood retinal barrier leakage. It was worth noting that blood retinal barrier leakage was decreased in association with the increase in ocular blood flow perfusion.

### 3.3. The Effect of *α*-Mangostin on Lipid Peroxidation, Advanced Glycation End Products, Receptor of Advanced Glycation End Products, Tumor Necrosis Factor Alpha, and Vascular Endothelial Growth Factor

**Figure 3:**
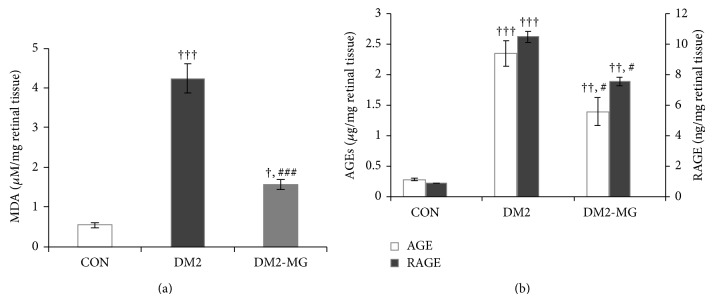
(a) Retinal malondialdehyde (MDA) and (b) advanced glycation end products (AGE_S_) and receptor of advanced glycation end product (RAGE), which were determined in normal control rats (CON), type 2 diabetic rats (DM2), and *α*-MG supplemented type 2 diabetic rats (DM2-MG). Values are presented as mean ± SEM. ^+,++,+++^ indicate significant differences compared to the CON rats at *P* < 0.05, *P* < 0.01, and *P* < 0.001, respectively. ^#,###^ indicate significant differences compared to the DM2 rats at *P* < 0.05 and *P* < 0.001, respectively.

**Figure 4:**
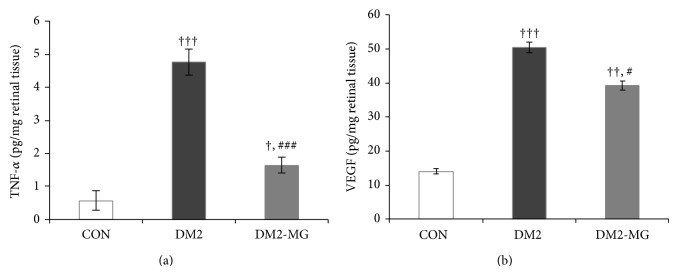
(a) Retinal tumor necrotic factor alpha (TNF-*α*) and (b) vascular endothelial growth factor (VEGF) which were determined in normal control rats (CON), type 2 diabetic rats (DM2), and *α*-MG supplemented type 2 diabetic rats (DM2-MG). Values are presented as mean ± SEM. ^+,++,+++^ indicate significant differences compared to the CON rats at *P* < 0.05, *P* < 0.01, and *P* < 0.001, respectively. ^#,###^ indicate significant differences compared to the DM2 rats at *P* < 0.05 and *P* < 0.001, respectively.

Changes in lipid peroxidation, advanced glycation end products, receptor of advanced glycation end products, tumor necrosis factor alpha, and vascular endothelial growth factor were also investigated. It was noted that the levels of those five parameters went to the same direction. In the DM2 rats, the retinal MDA, AGEs, RAGE, TNF-*α*, and VEGF levels were significantly higher than those determined in retinal tissue of the normal control rats. Interestingly, supplementation with *α*-MG gave good effects of treatment in DM2-MG rats, achieving about 63.2%, 40.9%, 27.8%, 65.6%, and 22.3% reductions in MDA, AGE, RAGE, TNF-*α*, and VEGF, respectively (Figures 3(a) and 3(b), Figures 4(a) and 4(b)).

## 4. Discussion

In the present study, we have demonstrated that alpha-mangostin supplementation is capable of improving the impairment of retinal microvasculature including ocular hypoperfusion and blood retinal barrier leakage in type 2 diabetic rat model induced by high fat diet with a low dose of STZ. The results are consistent with previous reports that the combination of high fat diet and a low dose of STZ administration (HFD-STZ) caused hyperinsulinemia and hyperglycemia [25, 29], resulting in the significantly increased HOMA-IR index in DM2 rats. Since HOMA-IR index has been used to predict insulin resistance, therefore, the insulin resistance should develop in our type 2 diabetic rats as well. Hyperinsulinemia found in the early stage of type 2 diabetes may be a result of compensatory mechanism of pancreatic *β*-cells in order to maintain blood glucose concentration at a normal level. In our study, although a low dose of STZ was used, the increased blood glucose (330–400 mg/dL) was developed. Such a low concentration of STZ (35 mg/kg) injection might partially damage the *β*-cells, leading to moderate hyperglycemia. Besides hyperglycemia and hyperinsulinemia, these HFD-STZ rats also showed hypercholesterolemia and hypertriglyceridemia, which are manifestations similar in human type 2 diabetes [30].

As shown in Figure 1 and Table 1, supplementation of *α*-MG for 8 weeks in DM2-MG rats could reduce the blood glucose, cholesterol, and triglyceride levels. These indicated that *α*-MG has antihyperglycemic and antihyperlipidemic effects. In addition, we found that *α*-MG treated rats showed low level of HOMA-IR, suggesting an interesting efficacy of *α*-MG to improve insulin resistance in type 2 diabetes. It is possible that a low dose of STZ could partially damage *β*-cells and left some cells surviving. Supplementation with *α*-MG can increase the insulin sensitivity and decrease the blood glucose level by improving the damaged *β*-cell functions and maintaining the survived *β*-cell functions. This conclusion is consistent with a recent study that oral feeding for two weeks of juice mangosteen rind could reduce hyperglycemia by repairing the damaged islet of Langerhans cells of STZ diabetic rats [31].

In an attempt to investigate whether the supplementation with *α*-MG could attenuate the retinal microvascular alterations in type 2 diabetic rats, we examined the effect of oral supplementation of *α*-MG on ocular blood flow and blood retinal barrier leakage. As shown in Figure 2(a), supplementation of *α*-MG increased OBF and decreased Evan blue dyes leakage.

The earliest signs of retinal microangiopathy in diabetes have been characterized as pericyte loss, leukocyte adhesion, endothelial cell death, and alterations in retinal blood flow [32, 33]. Previous studies have demonstrated the decreased retinal blood flow in both diabetic patients [34] and diabetic animal models [35]. Our present results confirmed the previous reports that reduction in ocular blood flow was developed in the early stage of diabetes mellitus. Several studies have proposed that reduced blood flow in diabetic eyes is associated with an increase in the expression of endothelial vasoconstrictor, endothelin-1 (ET-1) [36, 37]. Increasing vascular expression of ET-1 enhanced vascular resistance including in the retinal-vasculature, causing decreased retinal blood flow [38]. It is well recognized that hyperglycemia brings about changes in various biochemical pathways such as polyol pathway, nonenzymatic glycation, and diacylglycerol-PKC activation pathways. These alterations produced reactive oxygen species (ROS) which have been proposed to one of the major causes of hyperglycemia-induced endothelial dysfunction [39, 40]. The key features of endothelial dysfunction are not only a decrease in production and biological activity of potent vasodilator nitric oxide (NO) but also an increase in production of vasoconstrictor ET-1 [41]. Apart from the elevated expression of ET-1, adherence of leukocyte to retinal endothelium might be an important factor to lessen OBF in early diabetic retinopathy [42]. Miyamoto et al. have suggested that leukocyte adhesion had a great influence on retinal blood flow reduction in early stage of diabetic retinopathy [43]. Additionally, our previous study has revealed a significant increase in the number of adherent leukocytes in the iris microvessels which was associated with the reduction in its blood flow, investigated in STZ induced diabetic rats. However, these two events were improved by supplementation with antioxidant, vitamin C [44]. Therefore, it is indicated that hyperglycemia-induced oxidative stress modulated the overexpression of ET-1 and raised adherent leukocyte to vascular endothelium. These may result in the reduction of OBF in early stage of diabetic retinopathy. Furthermore, in our results it should be noted that DM2 rats showed the Evan blue dyes leaking from retinal vessels to parenchyma, indicating the blood retinal barrier (BRB) leakage. Interestingly, the data demonstrates the good correlation between OBF and BRB leakage as shown in Figure 2(b). It is indicated that in the early stage of diabetes the alteration of OBF may affect the integrity of BRB. Taken together, the present study has shown that oral supplementation with free radical scavenger, *α*-MG, for 8 weeks in DM2 rats could restore OBF and minimize the leakage of BRB as shown in Figure 2(a), which indicates a potential effect of *α*-MG in the attenuation of retinal microangiopathy in diabetes.

Alpha-MG is one of the major active compounds which is first isolated from the dried skin of mangosteen (*Garcinia mangostana *L.). Previous studies have demonstrated the anti-inflammatory and antioxidant effects of *α*-MG both in* in vitro* and* in vivo* [45, 46]. Therefore, we further looked for the mechanisms of *α*-MG involved in improvement of impaired retinal microvascular function in HF-STZ induced type 2 diabetes. It has been suggested that hyperglycemia and oxidative stress were the key events in the pathogenesis of retinal microangiopathy [47]. In addition, membrane lipid peroxidation and oxidative damage to DNA were elevated in the retina of diabetic rats [48]. In present study, we found significantly higher retinal malondialdehyde (MDA) levels in DM2 rats. In diabetes, there is an increase in free radicals production which in turn promotes lipid peroxidation. MDA is formed as an end product of lipid peroxidation, controlled by the antioxidants system under normal condition. Recently, Márquez-Valadez et al. demonstrated that *α*-MG exerts an antilipid peroxidative effect in brain tissue through its properties as a free radical scavenger [49]. In the present investigation, *α*-MG lessened lipid peroxidation shown by reducing the MDA levels in retinal tissues of DM2-MG rats compared with nontreated DM2 rats. Therefore, our result provides an evidence to show the antioxidant effect of *α*-MG in diabetes.

Oxidative stress in diabetes may be generated by several biochemical pathways, including nonenzymatic glycation end products (AGEs). In the present study, the increases in AGEs, RAGE, TNF-*α*, and VEGF levels were observed in the retinal tissues of DM2 rats. In addition, many evidences showed that AGEs induced oxidative stress generation in various types of cells by occurring via the interaction with its receptor (RAGE) and subsequently stimulated inflammation [50, 51]. Mohamed et al. have suggested that increasing of ROS might be produced through the AGEs-RAGE pathway, leading to the activation of the NF-*κ*B [52]. AGEs-RAGE and NF-*κ*B signaling have been reported to play a crucial role in increasing proinflammatory cytokines, such as TNF-*α* [53]. The increased ROS is also a great inducer for the release of TNF-*α* promoting to endothelial dysfunction in diabetes [54]. In the present study, we demonstrated a significant increase in TNF-*α* levels in retinal tissue of DM2 rats compared with those of age-matched normal control. Though TNF-*α* is known to increase retinal vascular permeability, its function could be inhibited by a soluble TNF receptor to prevent BRB breakdown in the diabetic rat model [55]. Increasing in vascular permeability caused by the breakdown of the blood retinal barrier is a hallmark of diabetic retinopathy. Yamagishi et al. suggested that AGE induced BRB dysfunction might be a result of stimulating retinal VEGF expression in diabetic rats [56]. In this point, we have shown that the levels of MDA, AGEs, RAGE, TNF-*α*, and VEGF in DM2-MG rats' retinas were significantly decreased after 8-week supplementation with *α*-MG compared with those in retinas of nonsupplemented DM2 rats. This would guide us to propose a possible mechanism by which *α*-MG suppresses BRB leakage in DM2 rats through the reduction in AGE-RAGE accumulation.

Vascular alterations in the early stage of DR include acellular capillaries, microaneurysms, and thickening of vascular basement membrane [57]. These vascular abnormalities are closely related to the BRB leakage. In addition, the loss of the neuroretinal cells may influence ocular hypoperfusion and oxidative injury [58]. These alterations occur in the early stage of DR. In this stage, vision loss is mainly caused by macular edema as the consequence of increased BRB permeability. For this reason, the present study focused on the effects of *α*-MG on restoration of OBF and an improvement of BRB integrity in type 2 diabetic rats. In 1965, Ashton suggested that early lesions of diabetic retinopathy were “focal breakdown of BRB” [59]. Then, Cunha-Vaz et al. showed that the alteration of BRB could be detected and measured in diabetic eyes with normal fundi using vitreous fluorometry [60]. Thereafter, many experimental and clinical studies have examined the alteration of the BRB in diabetes, suggesting that BRB breakdown in diabetic retina may have an important role in its development and progression of DR [61, 62]. Therefore, alteration of BRB evaluated by vitreous fluorometry or other methods, such as Evan blue dye technique, could be one parameter to investigate the early sign of diabetic retinal disease.

The present study investigated the efficacy of *α*-MG to prevent or delay diabetic-induced retinal microangiopathy and provided the first* in vivo* evidence of *α*-MG alleviating the early stage DR. However, a limitation of this study was that it did not demonstrate whether *α*-MG is able to protect rats from other developing vascular abnormalities, apart from OBF and BRB. Therefore, a further study related to important vascular changes, for example, acellular capillaries, microaneurysms, thickening of vascular basement membrane, and loss of retinal cells, should be conducted to support the therapeutic effect of *α*-MG in treating diabetic retinopathy.

## 5. Conclusion

The overall findings indicate that leakage of the BRB and reduction of OBF in HF-STZ induced type 2 diabetic rat model are associated with hyperglycemia and the accumulations of free radicals, AGEs-RAGE, TNF-*α*, and VEGF levels in the retinal tissues. Our findings provide the* in vivo* experiment that *α*-MG supplementation improves retinal microangiopathy in type 2 diabetic rats via its antihyperglycemic, antioxidant, anti-inflammatory, and antiglycation properties. Alpha-mangostin supplementation may be considered as an alternative choice used for the prevention of retinal microvascular complications in diabetic patient.

## Figures and Tables

**Table 1:** Mean arterial blood pressure (MAP), plasma glycated hemoglobin (HbA1C), serum insulin (S. insulin), homeostatic model assessment of insulin resistance (HOMA-IR), blood cholesterol (CHOL), and triglyceride (TG) levels in normal control rats (CON), type 2 diabetic rats (DM2), *α*-MG supplemented normal control rats (CON-MG), and *α*-MG supplemented type 2 diabetic rats (DM2-MG) at the 8th week supplementation.

	CON (*n* = 8)	CON-MG (*n* = 8)	DM2 (*n* = 7)	DM2-MG (*n* = 7)
MAP (mmHg)	104.79 ± 1.10	102.46 ± 1.46	129.95 ± 1.89^+++^	111.52 ± 1.95^+,###^
HbA1C (mg/dL)	3.73 ± 0.08	3.57 ± 0.04	6.70 ± 0.43^+++^	5.18 ± 0.32^++,#^
S. insulin (U/L)	4.37 ± 0.47	4.68 ± 0.50	155.00 ± 8.48^+++^	128.50 ± 8.71^+++,#^
HOMA-IR	1.20 ± 0.11	1.29 ± 0.30	154.80 ± 13.90^+++^	90.45 ± 6.82^+++,##^
CHOL (mg/dL)	96.20 ± 2.46	92.80 ± 1.86	156.14 ± 6.25^+++^	112.00 ± 3.24^+++,###^
TG (mg/dL)	90.70 ± 1.41	80.50 ± 1.02	136.43 ± 7.35^+++^	101.28 ± 4.17^###^

Values are presented as mean ± SEM.

ns: not significantly different from the CON rats.

^+,++,+++^indicate significant differences compared to the CON rats at *P* < 0.05, *P* < 0.01, and *P* < 0.001, respectively.

^#,##,###^indicate significant differences compared to the DM2 rats at *P* < 0.05, *P* < 0.01, and *P* < 0.001, respectively.
